# Machine learning-driven analysis and process optimization of FePt-BN granular films for HAMR applications

**DOI:** 10.1080/14686996.2026.2699076

**Published:** 2026-07-16

**Authors:** Daisuke Ogawa, Tetsuya Shoji, Masao Yano, Yusuke Matsuoka, Yukiko K. Takahashi

**Affiliations:** a Research Center for Magnetic and Spintronic Materials, National Institute for Materials Science, Tsukuba, Japan; b Advanced Materials Engineering Division, Toyota Motor Corporation, Susono, Japan; c Research Institute of Electrical Communication (RIEC), Tohoku University, Sendai, Japan

**Keywords:** FePt-BN granular films, process optimization, machine learning

## Abstract

The ever-increasing demand for high-density, energy-efficient data storage, propelled by AI and cloud infrastructures, is driving advancements in heat-assisted magnetic recording (HAMR) media. L1_0_-ordered FePt granular thin films are recognized as leading candidates owing to their exceptional thermal stability and potential for sub-10 nm grain sizes. Here, we investigate FePt-BN granular films, systematically optimizing their microstructure and magnetic properties by tuning process parameters including BN atomic fraction of FePt-BN targets, N_2_ gas flow, and film thickness. Employing a hybrid workflow that integrates conventional trial-and-error experimentation with machine learning such as principle component analysis, random forest regression and Bayesian optimization, we identify key determinants governing average main- and sub-grain size (*D*_1_, *D*_2_), coercivity (*μ*_0_*H*_c_), and chemical ordering. Machine-learning analysis reveals that the degree of L1_0_ order is the primary factor for *μ*_0_*H*_c_, while *D*_1_ is mainly influenced by BN content and microstructural attributes derived from transmission electron microscopy data. Active-learning-guided Bayesian optimization enabled us to rapidly achieve FePt-BN media with high *μ*_0_*H*_c_ (2.4 T), sub 6 nm grain diameters, and areal grain densities exceeding 12 Tgrains/in^2^ outperforming traditional heuristic approaches with fewer experiments. Our results underscore the power of data-driven process optimization for accelerated HAMR materials development, enabling the realization of next-generation hard disk drives with ultra-high recording densities.

## IMPACT STATEMENT

This study establishes a data-driven framework for understanding and optimizing nanostructured magnetic materials, using FePt-BN granular thin films for heat-assisted magnetic recording (HAMR) as a model system.

The central novelty lies in the integration of advanced data analytics with experimental materials science to extract physically meaningful structure–property relationships from complex, high-dimensional datasets. By combining principal component analysis of XRD and TEM data with machine-learning models, we demonstrate that coercivity is governed not simply by grain size, but more fundamentally by sub-grain structure and grain-boundary characteristics – providing new physical insight into magnetization stability in nanoscale systems.

Furthermore, this work introduces an active-learning-based experimental loop, in which Bayesian optimization directly guides thin-film synthesis, enabling rapid convergence toward optimal microstructures with significantly reduced experimental cost. This approach transforms conventional empirical process optimization into a systematic and reproducible methodology.

Beyond the specific FePt-BN system, the proposed framework is broadly applicable to a wide range of functional materials, offering a general strategy for accelerating materials discovery and design through the fusion of experimental techniques and data science.

## GRAPHICAL ABSTRACT

**Figure:**
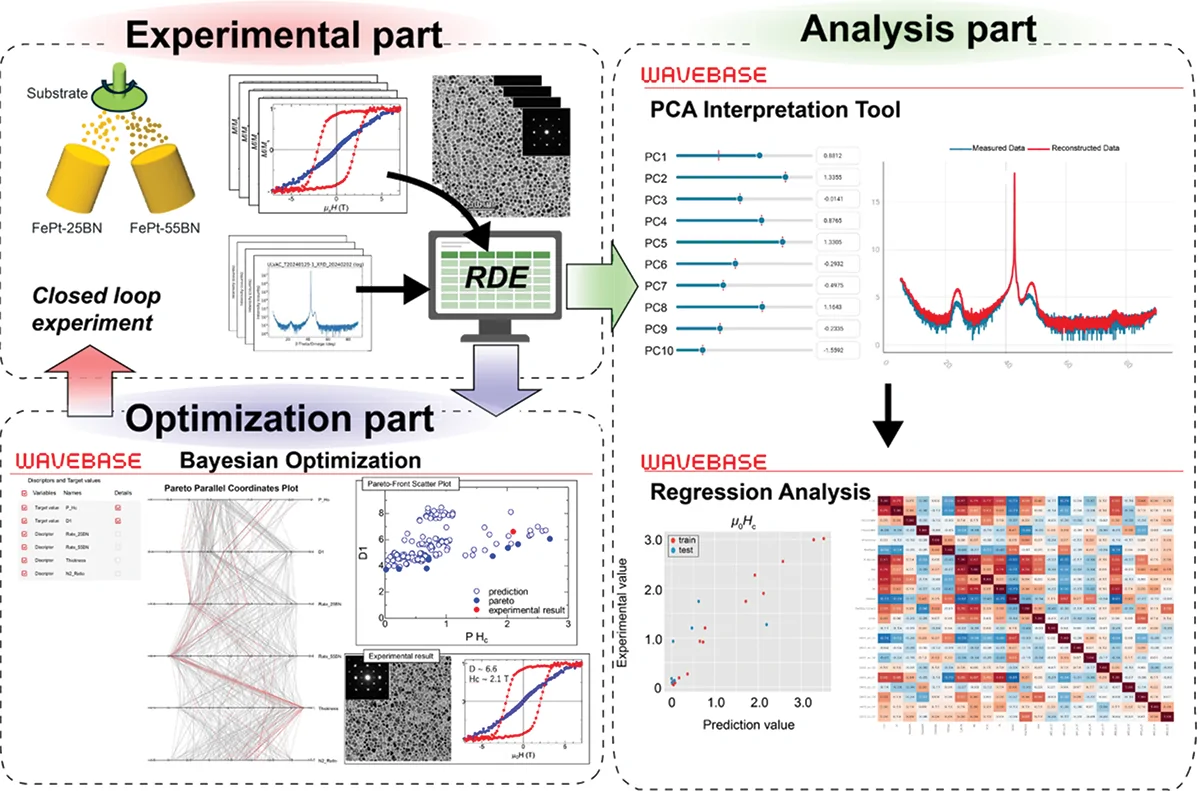
Schematic illustration of the active learning workflow employed for process optimization. FePt-BN granular films deposited by sputtering were characterized by SQUID, XRD, and TEM analyses. The resulting data were analyzed using the wavebase software platform, which performed principal component analysis (PCA), random forest regression, and Bayesian optimization to propose the next experimental conditions. This closed-loop process – comprising film deposition, measurement, machine learning-based data analysis, and feedback for experimental design – was iteratively repeated to efficiently optimize fabrication parameters toward improved FePt-BN granular microstructures and magnetic properties.

## 1. Introduction

The explosive growth of IoT, AI, and cloud services has intensified the demand for high-capacity, nonvolatility, and energy-efficient data storage, keeping hard disk drives (HDDs) indispensable in modern data centers. Heat-assisted magnetic recording (HAMR) [1–3] has therefore emerged as a leading technology to extend HDD areal density beyond the limits of conventional perpendicular magnetic recording by enabling reliable writing on media with substantially enhanced thermal stability. HAMR leverages localized, nanosecond-scale heating – typically using a laser and a near-field transducer – to momentarily reduce the effective switching field near the Curie temperature and then ‘freeze’ information upon rapid cooling, thereby reconciling writability and long-term stability at room temperature.

The underlying concept of thermally assisted writing has been explored for decades, yet practical implementation required a media material with exceptionally high magnetocrystalline anisotropy [4,5]. Chemically ordered L1_0_-FePt has been a premier candidate because its large anisotropy allows grain-size reduction while suppressing superparamagnetic degradation. In granular HAMR media, however, recording performance is governed not only by intrinsic magnetic properties but also by microstructural variability: grain size and its distribution, intergranular spacing, and the heat-spot geometry collectively drive transition noise and transition jitter. As areal density increases, the tolerance to dispersions in Curie temperature and anisotropy further tightens, motivating multilayer concepts such as exchange-coupled composite (ECC) structures [6,7] that broaden the operational window and mitigate thermal noise while also addressing reliability concerns associated with high-temperature writing.

A pivotal breakthrough toward manufacturable HAMR media was the discovery of FePt-C granular films [8], where amorphous carbon efficiently isolates grains and improves thermal stability. Building on such advances, Seagate initiated HAMR commercialization in 2020, demonstrating that the ecosystem of heads, near-field transducers, and media can be integrated at scale. Nevertheless, several challenges remain before broader deployment: ensuring write reliability under repeated thermal cycling, and reducing cost and yield losses associated with producing high-quality underlayers (e.g. MgO) required to achieve strong L1_0_ ordering and perpendicular texture. Solutions including ECC-HAMR [9,10] and alternative underlayer strategies (e.g. MgO-based templates, MTO) [11,12], and so on, continue to be actively developed.

In parallel with reliability and manufacturability, further areal-density scaling demands tighter control of the effective magnetic footprint and transition jitter, which are strongly coupled to the thermal gradient and the grain-size distribution. Multilevel recording concepts using multiple layers with different Curie temperatures – enabling controlled switching by tuning the heat-spot peak temperature – have been proposed as one route to higher density [13,14]. In this work, we instead focus on a materials-centric pathway: achieving higher areal density through grain-size and grain-shape control in FePt granular media. Roadmap analyses have long suggested that FePt-based HAMR has the potential to reach multi-terabit-per-square-inch densities if stringent microstructural and magnetic specifications are satisfied [15].

While FePt-C remains a benchmark, issues such as the formation of ‘secondary grains’ and limitations in attainable aspect ratio motivate exploration of alternative segregants [8]. Among various attempts to discover alternative segregant materials that can replace conventional C [16–18], boron nitride $\left(\mathrm{BN}\right)$ is particularly attractive because bias-assisted sputtering can induce the formation of *h*-BN nanosheets that conformally wrap FePt grains, promoting tall, well-isolated columnar structures while suppressing coalescence even at increased thickness [19–23]. Although promising, FePt-BN has comparatively few reports and its process window is not yet fully optimized.

Meanwhile, recent advances in artificial intelligence (AI) and machine learning (ML) have strongly accelerated data-driven materials research, including large-scale AI-assisted materials exploration [24] and closed-loop materials development that integrates ML, active learning, and automated or robotic experimentation [25]. Such closed-loop optimization has been applied to thin-film growth [26], composition-gradient experiments [27,28], high-entropy alloy discovery [29], optimization of direct extrusion process [30], and FePt-BNC nanogranular films [31] enabling efficient exploration of high-dimensional materials design spaces with limited experimental data. In addition, principal component analysis (PCA) provides an objective and statistically robust approach for extracting interpretable features from limited characterization datasets without subjective feature selection [32–34]. Although principal components do not necessarily have direct physical meaning, PCA-derived descriptors have been successfully correlated with crystallinity, texture, lattice strain, and phase fraction in X-ray diffraction patterns [35], as well as magnetization reversal, coercivity (*μ*_0_*H*_c_), and energy landscapes in magnetic domain images [36]. Thus, PCA is well suited for FePt-based granular media, where subtle variations in L1_0_ ordering, and grain morphology must be linked to key metrics such as *μ*_0_*H*_c_ and grain size.

Accordingly, this study aims to enable efficient optimization of FePt-BN granular media in a high-dimensional sputtering process space through a data-driven approach. The proposed framework consists of two main components. First, regression analysis was performed using sputtering process parameters and 10 PCA-derived components objectively extracted from X-ray diffraction (XRD) patterns and transmission electron microscopy (TEM) images, without relying on subjective human selection, as descriptors, with *μ*_0_*H*_c_ used as target variable. Second, Bayesian optimization was conducted using sputtering process parameters as descriptors to optimize FePt-BN granular media for heat-assisted magnetic recording (HAMR), with the objective of achieving both high *μ*_0_*H*_c_ and high grain density (small grain size).

## 2. Experimental details

### 2.1. Thin film preparation

FePt-BN granular thin films were deposited using an ultra-high-vacuum magnetron sputtering system with a base pressure of approximately 1 × 10^−7^ Pa. Single-crystal MgO(100) substrates [37] were used to promote the (001)-textured growth of chemically ordered L1_0_-FePt. The substrate temperature was set to 700°C during deposition, and the working pressure was maintained at 0.478 Pa in Ar with optional N_2_ addition, as described below. 700°C was chosen as it is the highest achievable with our setup and has yielded high L1_0_ ordering in previous studies [21]; optimization at higher temperatures remains future work.

The FePt-BN recording layers were deposited with nominal thicknesses in the range of 3.5–10 nm. The films were fabricated by simultaneous co-sputtering from 2-inch composite targets, FePt-25 at.% BN and FePt-55 at.% BN. The effective BN fraction in the deposited films was tuned by adjusting the DC power ratio applied to the FePt-25 at.% BN and FePt-55 at.% BN targets while keeping the substrate temperature and gas pressure constant. Specifically, the DC power of each target was varied from 0 to 100 W to span a broad process window for microstructural control. In addition, the N_2_ flow rate was set to 0, 2.5, or 5.0 sccm (with the total pressure fixed) to assess the influence of nitrogen incorporation on BN-related bonding states and on the formation of well-isolated granular structures. To suppress surface oxidation during exposure to air and subsequent characterization, a 5 nm thick amorphous carbon capping layer was deposited at room temperature immediately after FePt-BN deposition.

### 2.2. Characterization

Crystal structure and L1_0_ chemical ordering were evaluated by X-ray diffraction (XRD) using a SmartLab diffractometer equipped with a Cu-Kα source (Rigaku Corporation, Japan). The L1_0_ ordering parameter *S* was quantified from the integrated intensities of the (001) superlattice and (002) fundamental reflections, following the same analysis procedure as in our previous reports for highly (001)-textured FePt granular films [10].

The chemical bonding state associated with B–N in the FePt–BN granular films was examined by X-ray photoelectron spectroscopy (XPS) using a Quantera SXM system (ULVAC-PHI, Inc., Japan), which has a proven track record as a powerful analytical tool [23]. All spectra were calibrated to the C 1s peak at 284.8 eV [38]. Background subtraction was performed using the Shirley method [39], and the integral intensities of relevant peaks were obtained by Pseudo-Voigt fitting to enable quantitative comparisons among samples prepared under different sputtering conditions.

Microstructural characterization was performed by plan-view and cross-sectional scanning transmission electron microscopy (STEM) using TITAN and Spectra Ultra S/TEM instruments (Thermo Fisher Scientific, the Netherlands). Plan-view TEM lamellae were prepared by chemical etching, whereas cross-sectional lamellae were fabricated by focused ion beam (FIB) lift-out using a Helios 5 UX dual-beam SEM/FIB system (Thermo Fisher Scientific, USA). These observations were used to evaluate average main- and sub-grain size (*D*_1_, *D*_2_), pitch distance (*PD*), roundness, density, and pack fraction parameters were quantitatively evaluated from planar TEM images using an automated analysis workflow, as described in Ref. [40] as a function of BN fraction and N_2_ flow.

Out-of-plane and in-plane magnetic hysteresis loops were measured in magnetic fields of up to 7 T using a superconducting quantum interference device (SQUID) magnetometer (Quantum Design, USA) in magnetic fields up to 7 T.

### 2.3. Machine learning analysis

For data-driven process analysis and optimization, the XRD profiles, in-plane TEM images and M-H curves measured by SQUID were further analyzed using WAVEBASE [41–43]. The accumulation of XRD patterns, TEM images, and SQUID data, as well as the creation of datasets for machine learning analysis from these data, was carried out using Research Data Express (RDE), the cloud-based data acquisition system developed by NIMS [44,45]. PCA was applied to compress the high-dimensional descriptors derived from XRD patterns and TEM image features, and random forest regression analysis [46] was employed to explore influential process parameters and structure–property relationships in an interpretable manner.

Bayesian optimization [47,48] was then implemented in an active-learning loop using a Gaussian-process–based surrogate model. An initial dataset generated by human-guided trial-and-error experiments was used to initialize the surrogate model, which proposed subsequent deposition conditions by balancing exploration and exploitation. A film was fabricated and characterized under the proposed conditions, and the resulting data were appended to the dataset for the next optimization cycle. This iterative workflow was repeated to efficiently navigate the multi-parameter space and converge toward process conditions yielding improved FePt-BN granular microstructures and magnetic performance.

## 3. Results and discussions

### 3.1. Experimental part

Using an intuition-guided trial-and-error (heuristic) optimization approach, we optimized four process parameters – the sputtering powers for the FePt-25 at.% BN and FePt-55 at.% BN targets, the film thickness, and the $N_{2}$ gas flow rate – within 26 experimental iterations described in Table S1. The resulting best-performing condition, which simultaneously yields a smaller *D*_1_ and a higher *μ*_0_*H*_c_, is summarized in Figure 1(a-d). Figure 1(a,b) show the plan-view TEM image and the corresponding selected-area electron diffraction (SAED) pattern, respectively; Figure 1(c) shows the cross-sectional TEM image; and Figure 1(d) presents the out-of-plane and in-plane magnetization hysteresis loops.　From Figure 1(a), a well-isolated granular microstructure is observed. The corresponding diffraction pattern in Figure 1(b) exhibits superlattice reflections attributable to the L1_0_ ordering. As shown in Figure 1(c), the FePt grains form a columnar structure without secondary grains, and the magnetic hysteresis loops in Figure 1(d) indicate pronounced perpendicular magnetic anisotropy. Quantitative values extracted from the raw data are as follows: the in-plane average grain diameter *D* is 6.4 nm, the pitch distance *PD* is 6.9 nm, the areal density is 9.9 T/in^2^, the chemical order parameter *S* is 0.97, and the *μ*_0_*H*_c_ is 2.1 T.

**Figure 1.**
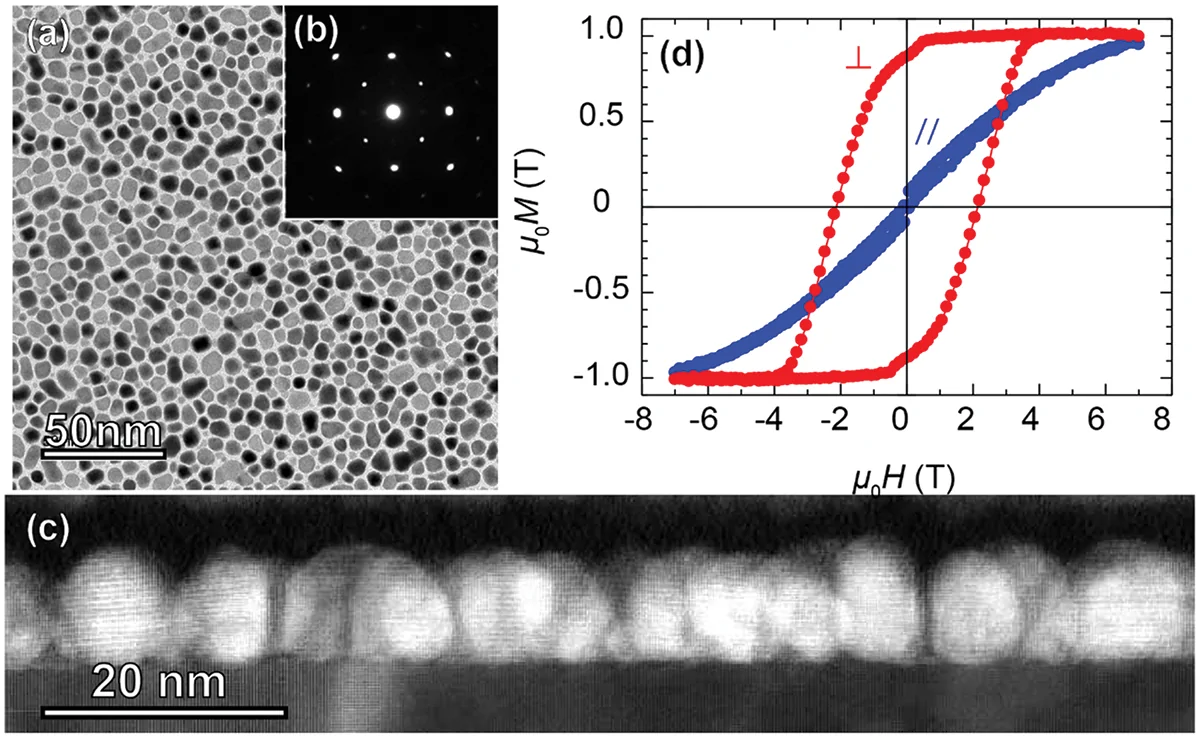
(a) Plan-view TEM image, (b) corresponding selected-area electron diffraction (SAED) pattern, (c) cross-sectional TEM image, and (d) out-of-plane and in-plane magnetization hysteresis loops of FePt-BN granular films fabricated with FePt-25 at.% BN and FePt-55 at.% BN targets at input powers of 10 W and 0 W, respectively, using a N_2_ gas flow of 10 sccm and a nominal thickness of 6 nm.

Next, we examined the influence of each experimental parameter on the resulting microstructure and magnetic properties. Figure 2 shows plan-view TEM images and magnetization hysteresis loops of 3.5 nm-thick FePt-BN thin films deposited under different N_2_ gas flow rates. Compared with the film prepared without N_2_ flow, introducing 5 sccm N_2_ leads to grain coarsening, accompanied by partial intergrain connectivity between adjacent FePt grains. With a further increase to 10 sccm, the *D*_1_ decreases again. In contrast, the *μ*_0_*H*_c_ decreases monotonically with increasing N_2_ flow rate. In addition, the in-plane hysteresis loops progressively evolve from a nearly linear response to a more pronounced *S*-shaped curvature as the N_2_ flow is increased.

**Figure 2.**
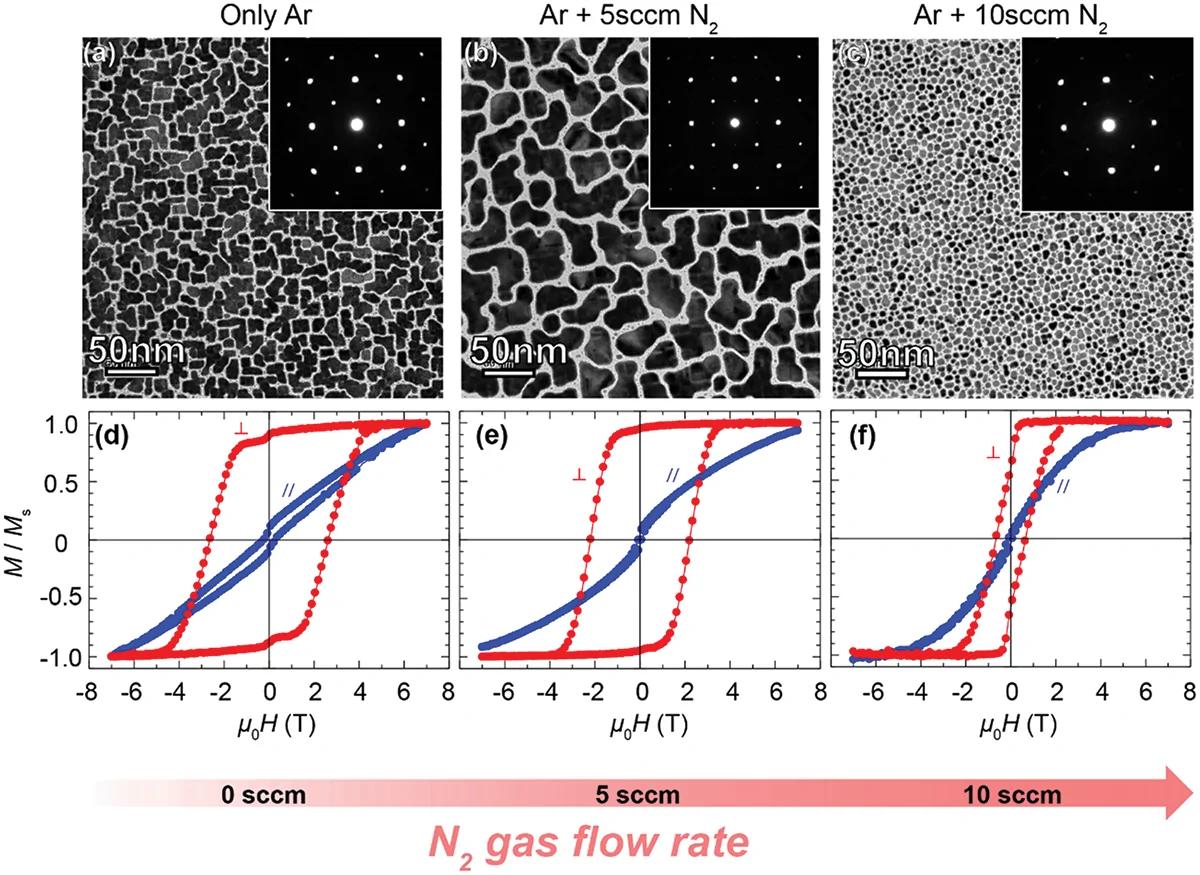
Plan-view TEM images and magnetization hysteresis loops (out-of-plane and in-plane) of FePt-BN thin films deposited under different N_2_ gas flow rates: (a), (d) without N_2_ gas; (b), (e) with 5 sccm N_2_ gas; and (c), (f) with 10 sccm N_2_ gas, respectively.

Figure 3 presents the B 1s and Fe 2p XPS spectra of these films. For all samples, the B 1s spectra can be deconvoluted into components associated with sp^3^-bonded BN and sp^2^-bonded BN [23,49]. Notably, as the N_2_ flow rate increases, the sp^2^-BN (*h*-BN-like) component becomes more prominent, indicating enhanced formation and segregation of *h*-BN at higher N_2_ supply [23]. The Fe 2p spectra (both Fe 2p_1/2_ and Fe 2p_3/2_), after fitting, display multiple components previously attributed to Fe^3+^ (tentatively assigned to Fe–N bonding), Fe^2+^ (tentatively assigned to Fe–B bonding), and metallic Fe (Fe–Pt) bonding states [50,51]. Upon increasing the N_2_ flow rate, we observe a decrease in the Fe^3+^ component intensity (‘Fe–N-like’) and a corresponding increase in the Fe^2+^ component intensity (‘Fe–B-like’). While this trend might initially appear internally inconsistent with the enhancement of *h*-BN formation (sp^2^-BN), we interpret the results as follows: higher N_2_ flow promotes the incorporation of nitrogen into B–N bonds, facilitating segregation and stabilization of BN as a distinct *h*-BN phase in the grain boundary. This reduces the availability of nitrogen to form Fe–N bonds at the FePt/BN interface, leading to diminished Fe–N-like (Fe^3+^) species at the interface. The concomitant rise in the Fe–B-like (Fe^2+^) signal may reflect a relatively higher prevalence of B-rich local bonding at the interface as BN segregation proceeds. We note that Fe 2p XPS peak deconvolution is sensitive to both bonding and local coordination/multiplet effects, so these assignments are necessarily approximate; thus, we now refer to these as ‘Fe–N-like’ and ‘Fe–B-like’ environments, consistent with the local bonding changes at the interface rather than direct quantification of bond populations. Overall, the XPS results support that with increasing N_2_ flow, N atoms are preferentially incorporated into B–N networks forming *h*-BN segregation phases, rather than into Fe–N bonds at the FePt interface.

**Figure 3.**
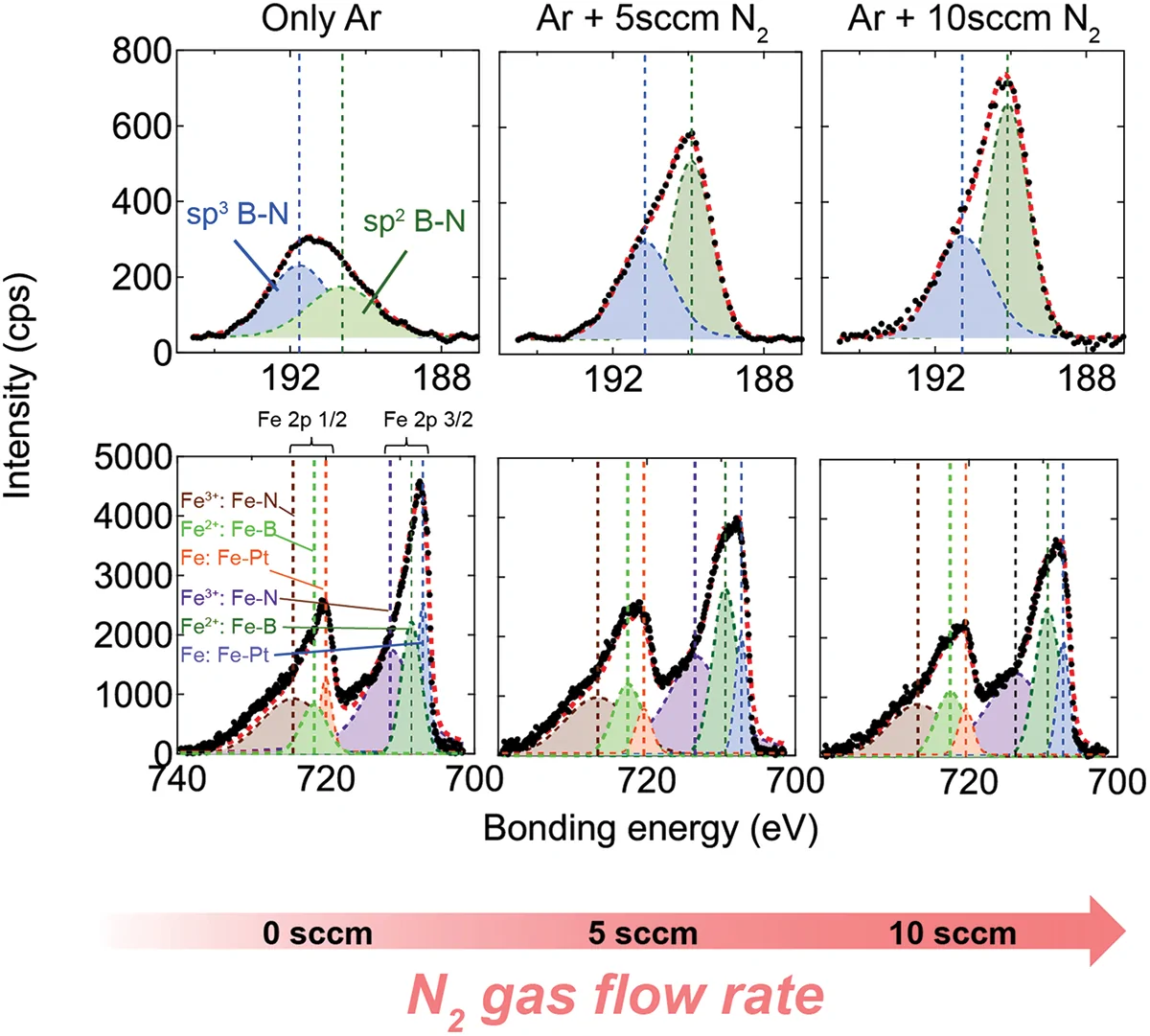
B 1s and Fe 2p XPS spectra of FePt-BN granular films prepared under different N_2_ gas flow rates: without N_2_ gas, with N_2_ gas flow of 5 sccm, and with N_2_ gas flow of 10 sccm.

Figure 4 summarizes the combined effects of BN atomic fraction of FePt-BN targets (vertical axis) and FePt-BN layer thickness (horizontal axis) by presenting, for each condition, the plan view TEM image, the corresponding selected-area electron diffraction (SAED) pattern, the cross-sectional TEM image, and the magnetic hysteresis loop. The thickness dependence clearly varies with BN atomic fraction.

**Figure 4.**
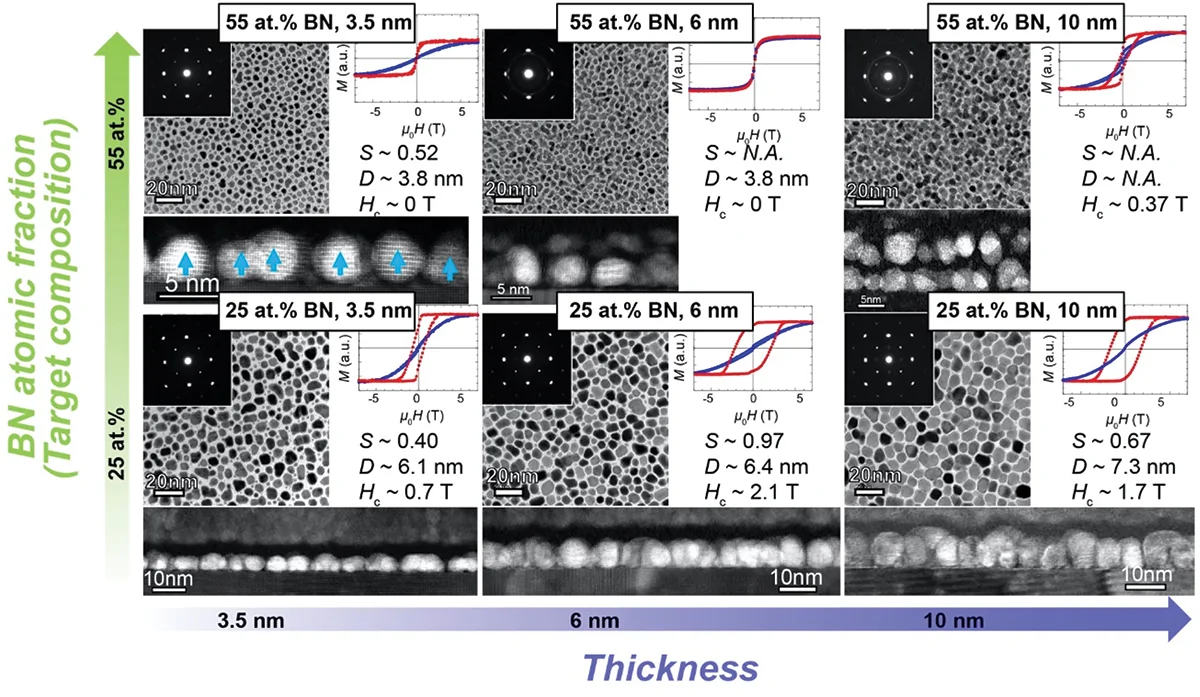
Summary of the combined effects of BN atomic fraction of target (25–50 at.%, vertical axis) and nominal thickness of FePt-BN layer (3.5–10 nm, horizontal axis) on the microstructure and magnetic properties of FePt-BN granular films. For each combination of BN fraction and film thickness, the plan-view TEM image, the corresponding selected-area electron diffraction (SAED) pattern, the cross-sectional TEM image, and the magnetic hysteresis (M-H) loop are presented. The dependence of layer thickness on film properties varies significantly with the BN atomic fraction.

For FePt-25 at.% BN films, the *D*_1_ increases slightly with increasing thickness. At 3.5 nm, an isolated-grain morphology is observed. When the thickness is increased to 6 nm, the in-plane *D*_1_ remains nearly unchanged (from 6.0 to 6.4 nm), whereas the out-of-plane aspect ratio increases, indicating preferential vertical grain growth. In parallel, the *μ*_0_*H*_c_ increases from 0.7 T to 2.1 T, which is attributed to an increase in the chemical ordering parameter *S* from 0.4 to 0.95. Upon further increasing the thickness to 10 nm, partial intergrain connections appear near the top region of the grains. The reduction in *μ*_0_*H*_c_ to 1.8 T is consistent with the decrease in *S* to 0.67, and the emergence of intergrain contacts in the top region is also likely to contribute to the *μ*_0_*H*_c_ degradation.

In contrast, for FePt-55 at.% BN films, a finer isolated nanogranular structure is formed at 3.5 nm compared with the FePt-25 at.% BN films. Although this film exhibits perpendicular magnetic anisotropy with *S* = 0.51, the *μ*_0_*H*_c_ is essentially zero at 300 K. This behavior is attributed to superparamagnetism: it is often suggested that superparamagnetic behavior emerges when *K*_u_*V*/*k*_B_*T*≤ 25 [52], and in the present case, the theoretical critical limit of superparamagnetism at 300 K is considered to be 3.7 nm for bulk FePt (*K*_u_ = 4 × 10^7^ erg/cm^3^) [4] or more larger (~5 nm) in the case of nanogranular films due to smaller *K*_u_ [53], assuming a spherical grain (*V* = (π/6)*D*^3^). Notably, this sample exhibits a *μ*_0_*H*_c_ of approximately 2 T at 50 K shown in Figure S1, supporting the above interpretation.

The best *D*_1_ (areal density) reported to date for FePt-based HAMR-type granular media is approximately 3.8 nm (corresponding to $4\mathrm{Tbit}/in^{2}$) when Cr_*x*_O_1-*x*_ is used as a segregant [54]; however, the literature also reports that *μ*_0_*H*_c_ becomes nearly zero at this grain size because the L1_0_ structure is not preserved. In the present study, an L1_0_ structure is obtained even at a comparable grain size, indicating that this limitation has been overcome. These results suggest that achieving both 4Tbit/in^2^ and high *μ*_0_*H*_c_ may be feasible if the aspect ratio and grain volume can be controlled via thickness while maintaining the in-plane grain size. Therefore, continued process optimization of FePt-BN granular films remains a viable and promising development pathway.

For BN = 55 at.%, increasing the thickness to 6 nm led to the formation of secondary-grain-like structures in the grain-top region, as clearly observed in the cross-sectional TEM image, and the *μ*_0_*H*_c_ remained nearly zero. This behavior is in contrast to that of the 25 at.% BN films, in which increasing the thickness from 3.5 to 6.0 nm enhanced L1_0_ ordering and increased *μ*_0_*H*_c_. In the 55 at.% BN films, the high BN content appears to destabilize the continuous columnar growth of FePt grains as the film thickness increases. At approximately 6 nm, the upper part of the film no longer maintains a well-defined columnar granular structure, and secondary grains form on top of the lower granular layer. This structural disorder likely degrades the perpendicular magnetic anisotropy, leading to nearly identical out-of-plane and in-plane hysteresis loops and negligible *μ*_0_*H*_c_. At 10 nm, the secondary grains grow more prominently, the FePt grains are clearly separated into lower and upper regions, and neighboring grains also begin to connect laterally in the film plane. These results indicate that excessive BN content combined with increased thickness disturbs the columnar granular morphology required for robust perpendicular magnetic anisotropy.

Overall, these results demonstrate that both the microstructure and *μ*_0_*H*_c_ are highly sensitive to thickness, and that this thickness dependence changes drastically with BN atomic fraction. As exemplified by the combined effects of thickness and BN atomic fraction, process parameters interact with each other and collectively govern grain morphology, L1_0_ ordering, and *μ*_0_*H*_c_. In such a complex system, machine-learning-based analysis can serve as a useful tool. In the next section, we analyze how process parameters influence *D*_1_ and *μ*_0_*H*_c_ using machine learning and present an optimization strategy that proposes subsequent experimental conditions.

In several of the FePt-BN films prepared in this study, distinct microstructural features were observed. Representative high-angle annular dark field (HAADF) and low-angle annular dark field (LAADF) STEM images illustrating such characteristic structures are presented in Figure 5(a,b), respectively.

**Figure 5.**
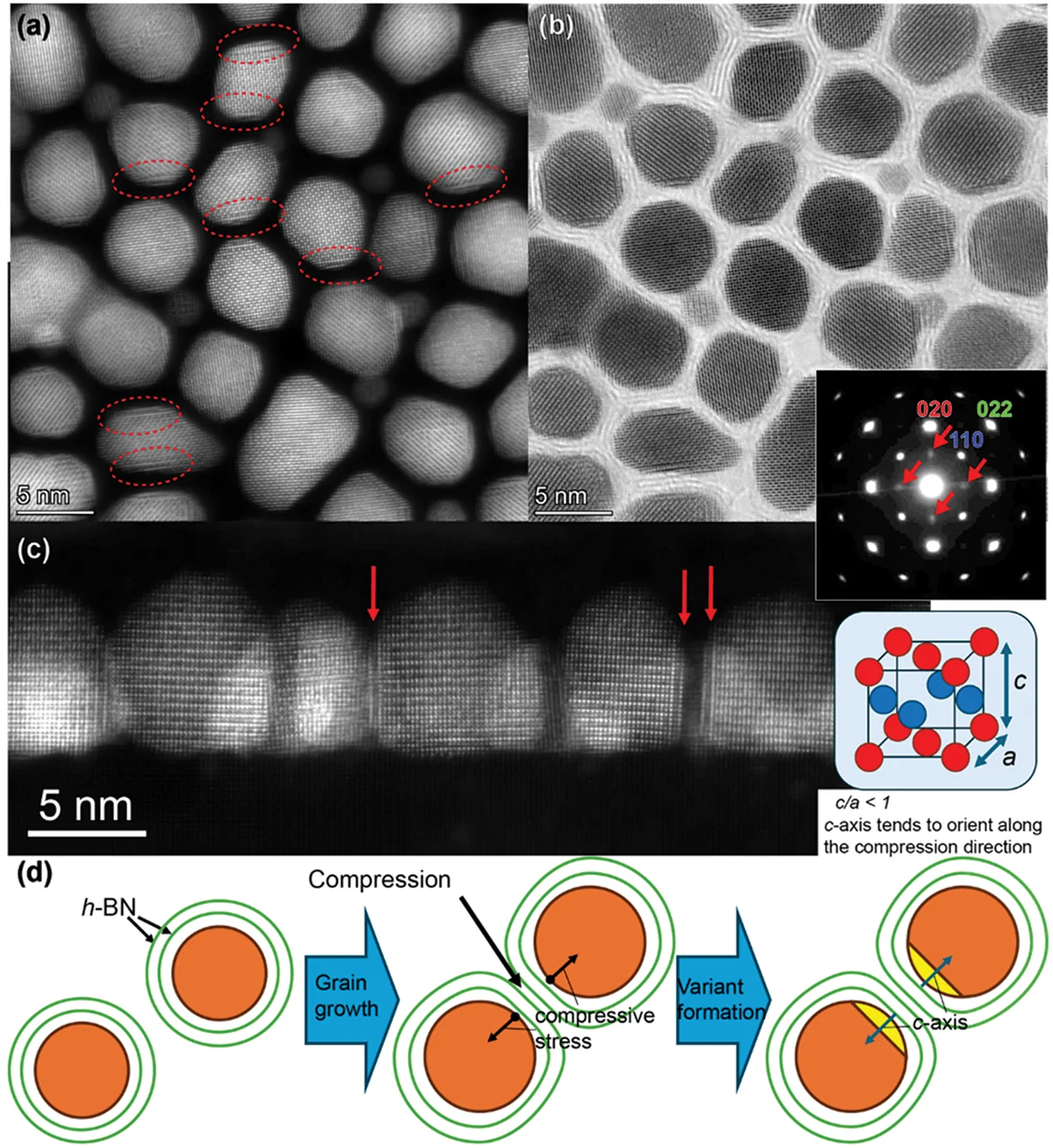
Scanning transmission electron microscopy (STEM) images of the FePt-BN granular film: (a) high-angle annular dark field (HAADF) image, (b) low-angle annular dark field (LAADF) image, the inset shows the correspond SAED pattern for FePt-BN granular film, (c) cross-sectional STEM image and (c) schematic illustration of mechanism for the formation of in-plane variants of FePt grains, representing how compressive strain in the interlayer *h*-BN may induce reorientation of the FePt *c*-axis parallel to the film plane.

As seen in Figure 5(b), *h*-BN with a layered structure is formed around and between the FePt nanograins, appearing as regions of darker contrast. From Figure 5(a), it is evident that the *c*-axes of the FePt grains are generally oriented perpendicular to the film plane. Notably, however, in certain regions where FePt is in contact with *h*-BN – highlighted by red dashed lines – clear superlattice fringes, originating from the alternating stacking of Fe and Pt atomic layers characteristic of the L1_0_-type structure, can be observed. This indicates that in these areas, the FePt [001] direction is oriented parallel to the film plane. In addition to the HAADF-STEM image, SAED pattern shown as the inset of Figure 5(b) has broad diffraction peaks, which is remarked with red arrows, originating from the in-plan variants. To further support this observation, additional cross-sectional STEM images in Figure 5(c) confirmed the presence of L1_0_-FePt regions with the *c*-axis oriented in the film plane at the edge of grains. These images demonstrate that the observed contrast is not solely due to step-like grain morphology or overlap with *h*-BN.

Although the origin of the in-plane variants cannot be definitively identified at this stage, three plausible factors can be considered. First, elastic strain associated with lattice mismatch has been suggested to influence both vertical grain growth and variant selection, because strain-energy relaxation favors microstructures that reduce the internal stress for the case of L1_0_-FePt with *c*/*a* < 1 [55]. During FePt grain growth, compressive stress exerted on the interlayer *h*-BN normal to the *h*-BN layers may, if sufficiently large, promote reorientation of the FePt *c*-axis parallel to the film plane, as schematically illustrated in Figure 5(d). Second, reduced epitaxial registry with the MgO underlayer at the edges of FePt grains, where contact with the underlayer is limited, could facilitate the formation of in-plane variants [56]. Third, orientation variants may also form to accommodate lattice mismatch and minimize the interfacial energy between FePt and *h*-BN.

### 3.2. Machine learning analyses

Figure 6 presents a portion of the heatmap showing the correlation coefficients among the process parameters and output variables for the 27 samples listed in Supplementary Table S1 (i.e. the sample set obtained after a heuristic optimization approach). In this analysis, the variables include *D*_1_ and *μ*_0_*H*_c_ as the target output variables and the process parameters: the deposition rates for FePt-25 at.% BN and FePt-55 at.% BN targets, the film thickness, and the N_2_ gas concentration. The results indicate that the correlation coefficient between *μ*_0_*H*_c_ and N_2_ ratio is −0.55, reflecting a relatively strong negative correlation that is consistent with the trend observed in Figure 2. In contrast, the correlation coefficient between *D*_1_ and the N_2_ ratio is −0.19, which is weakly negative and reflects the nonlinear relationship between *D*_1_ and N_2_ flow rate shown in Figure 2. This highlights that a simple linear correlation does not fully capture the complexity of this relationship. It should be noted that, due to the limited number of N_2_ ratio data points, the observed relationship should be regarded as a qualitative trend rather than a statistically significant correlation. Further data points are necessary to confirm any definitive relationship. Notably, ‘Roundness’ had the strongest correlation with *μ*_0_*H*_c_ among the examined morphological parameters, suggesting that it reflects not only grain circularity but also grain isolation and the absence of lateral interconnection with neighbouring grains. This relationship is physically reasonable because lateral connectivity and irregular grain shape in FePt granular films can affect intergranular exchange coupling and the coercive-field distribution. The correlation coefficient between *μ*_0_*H*_c_ and *D*_2_ is also relatively strong, which would derive that the existence of smaller sub grain leads to decrease *μ*_0_*H*_c_. However, we should care that ‘Roundness’ and ‘*D*_2_’ are unlikely to solely determine *μ*_0_*H*_c_; rather, it may covary with microstructural factors, including grain-boundary formation, grain isolation, L1_0_ ordering, and *c*-axis orientation, which collectively influence *μ*_0_*H*_c_.

**Figure 6.**
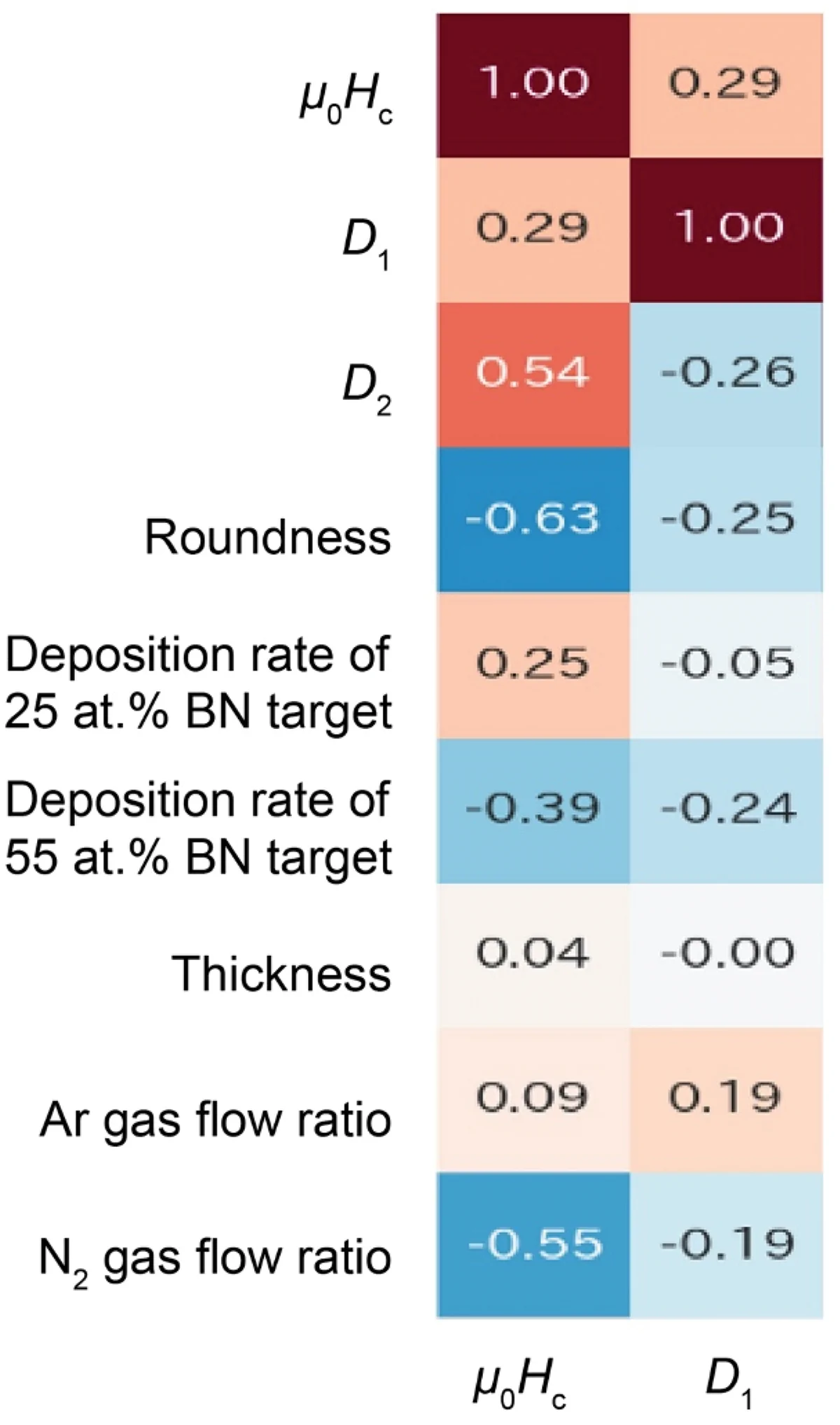
Heatmap of correlation coefficients among process parameters, structural descriptors, and magnetic properties for the FePt–BN granular films. The correlations indicate linear associations within the analyzed dataset and are intended for exploratory analysis rather than to establish causal relationships.

Next, we focused exclusively on *μ*_0_*H*_c_ as the target variable. In addition to the process parameters (the deposition rates for FePt-25 at.% BN and FePt-55 at.% BN targets, the film thickness, and the N_2_ gas concentration), we performed PCA on the XRD patterns and plan-view TEM images. PCA was adopted in this study to ensure statistical robustness and physical interpretability under the limited dataset size, rather than to maximize representational capacity. Given the small number of samples, more complex nonlinear dimensionality-reduction techniques, such as neural-network–based autoencoders, were considered less suitable due to the risk of overfitting and reduced interpretability. The PCA-derived components therefore serve as compressed descriptors that capture dominant structural variations in XRD and TEM data while remaining amenable to physical interpretation.

Applying PCA to high-dimensional XRD and TEM datasets enables effective dimensionality reduction, transforming complex, correlated descriptors into a compact set of orthogonal components. This dimensionality reduction facilitates subsequent machine-learning model construction and correlation analysis by mitigating multicollinearity and improving numerical stability, while preserving the dominant structural variations present in the original data.

For plan-view TEM images, PCA was performed on 1D power spectra obtained by radially integrating the 2D Fourier transforms, enabling extraction of dominant microstructural features for machine-learning analysis, in accordance with the workflow described in Ref [47]. These PCA-derived components were also included as input features in a random-forest regression model. Figure 7 shows the resulting parity plot (predicted vs. experimental) and the feature-importance ranking. The PCA component spectra for XRD and TEM are provided in Supplementary Figs. S3 and S4, respectively. We should note that the regression model showed strong performance on the training data (RMSE = 0.23, R^2^ = 0.96) but poor generalization to the test set (RMSE = 0.71, R^2^ = 0.08), indicating substantial overfitting. This discrepancy likely stems from the small dataset size (*N* = 35), experimental noise, and the inherent complexity of the underlying phenomena. Here, the RMSE values for *μ*_0_*H*_c_ are given in units of tesla (T). We emphasize that the random forest model was not used as a surrogate model for Bayesian optimization. Rather than relying on deterministic point predictions, the Bayesian optimization framework employed a Gaussian-process–based probabilistic surrogate to guide sample-efficient experimental exploration under severe data limitations.

**Figure 7.**
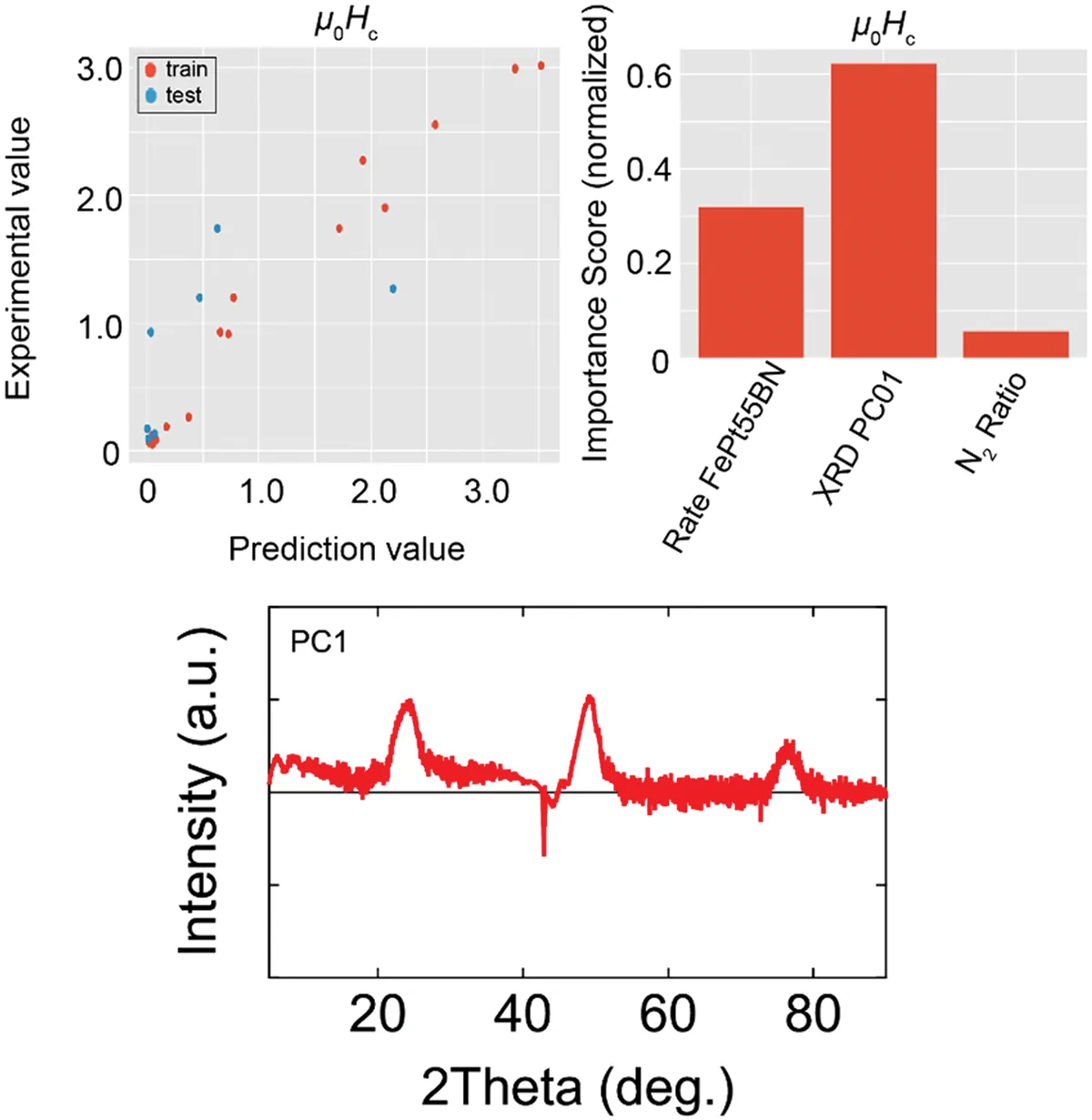
Parity plot (predicted vs. experimental values), feature-importance ranking, and the principal component PC01 of the XRD pattern, which exhibited the highest feature-importance ranking for *μ*_0_*H*_c_, for the FePt-BN granular films.

The model indicates that *μ*_0_*H*_c_ is most strongly governed by XRD PC01. Inspection of the PC01 loading spectrum reveals that it closely matches the characteristic diffraction features of L1_0_-FePt, suggesting that PC01 predominantly reflects the degree of L1_0_ crystallinity/ordering, as expected. Notably, PC01 also shows increased intensity at low 2θ angles (<20°), as shown in Figure S3. This region can include contributions from nanoscale microstructural inhomogeneity, such as interfaces, grain-boundary regions, or variations in interparticle connectivity. Therefore, the simultaneous strengthening of both the L1_0_-related diffraction features and the low-angle intensity in PC01 suggests that higher *μ*_0_*H*_c_ values are associated not only with enhanced L1_0_ ordering but also with microstructural features related to the granular morphology. However, because the low-angle signal can originate from multiple structural factors, we do not assign it to a single specific microstructural feature.

To further examine which microstructural descriptors are correlated with *μ*_0_*H*_c_, we constructed a regression model using only the principal-component features derived from TEM images, which encode information related to *D*_1_, *D*_2_, spacing, morphology, and interfacial contrast. Figure 8 presents the corresponding parity plot and feature-importance analysis, revealing that TEM PC01, PC05, and PC09 contribute most strongly to *μ*_0_*H*_c_, in this order. The RMSE and R_2_ values for the test data were 0.91 and 0.46, respectively, while those for the training data were 0.41 and 0.86, reflecting modest generalization performance due to the limited dataset size. Therefore, this analysis should be regarded as exploratory and hypothesis-generating rather than as a definitive predictive model or direct proof of a physical mechanism.

**Figure 8.**
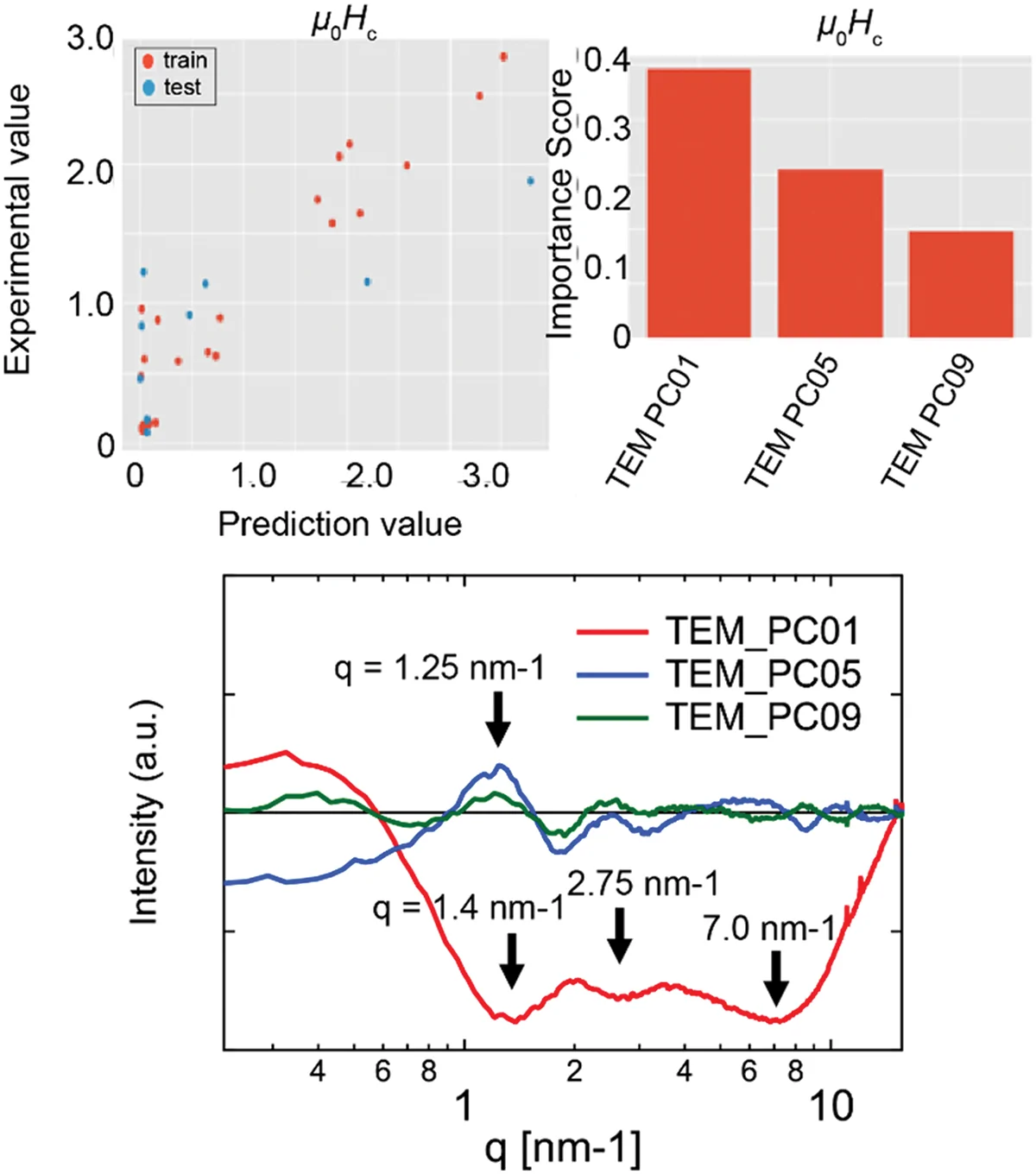
Parity plot and feature importance analysis for *μ*_0_*H*_c_, demonstrating that TEM features PC01, PC05, and PC09 have the greatest impact, in that order. The bottom panel shows the spectra corresponding to PC01, PC05, and PC09.

For TEM PC01, the loading spectrum exhibits an overall negative trend with pronounced features at *q* = 1.4, 2.75, and 7.0 nm^−1^, corresponding to real-space length scales of *d* = 4.5, 2.3, and 0.9 nm, respectively. These length scales are smaller than, or comparable to, the characteristic FePt grain size and may reflect fine structural features within or between grains, including local contrast variations, interfacial regions, or grain-boundary-related features. In addition, TEM PC01 contains a signal in the low-q region, *q* < 0.4 nm^−1^, corresponding to long real-space periodicities above 15 nm. This indicates that PC01 does not represent a single microstructural factor, but rather contains mixed information on both short- and long-range structural variations. In contrast, TEM PC05 shows a feature around *q* = 1.25 nm^−1,^ corresponding to *d* = 5.03 nm, which is close to the characteristic scale of the main FePt grains. However, PC05 also contains signals in both the low-*q* and shorter-period regions. Thus, it is not appropriate to simply assign TEM PC01 to ‘sub-grain structure and grain boundaries’ and TEM PC05 to ‘main grain size’.

These results indicate that the TEM-derived descriptors that correlate with *μ*_0_*H*_c_ are not limited to the *D*_1_ but include mixed structural information related to grain morphology, interparticle connectivity, and local interfacial contrast. Physically, such features can affect *μ*_0_*H*_c_ because magnetization reversal in granular FePt films is influenced not only by the intrinsic magnetocrystalline anisotropy associated with L1_0_ ordering but also by the degree of magnetic isolation between grains, intergranular exchange coupling, and the distribution of local switching fields. Nevertheless, the present PCA-based analysis does not by itself identify a unique microscopic origin of *μ*_0_*H*_c_. The more appropriate conclusion is that the structural information captured by TEM PC01 correlates more strongly with *μ*_0_*H*_c_ than that captured by TEM PC05, suggesting that microstructural features beyond the *D*_1_ are important descriptors for *μ*_0_*H*_c_.

In addition, Supplementary Figure S8 indicates that *D*_1_ is most influenced by (i) XRD PC05, (ii) the deposition rate of the FePt-25 at.% BN target, corresponding to the BN atomic fraction, and (iii) TEM PC03, in decreasing order of importance. For *D*_2_, the dominant factors are (i) the deposition rate of the FePt-25 at.% BN target and (ii) XRD PC01, again in decreasing order of importance. Although the discussion above presents only representative examples, the present analysis enables systematic identification of relationships among the examined process, structural, and magnetic parameters. A heatmap summarizing all parameters is provided in Figure S7. The loading spectra from the PCA of the XRD and TEM datasets are shown in Figs. S3 and S4, respectively, and the corresponding PCA scores for each sample are summarized in Table S2. To facilitate interpretation of the physical meaning of each principal component, comparisons between the minimum- and maximum-score cases are presented for XRD in Figure S5 and for TEM in Figure S6. These supplementary results provide additional information for interpreting the PCA components, although the components should be understood as mixed descriptors rather than as uniquely assigned structural quantities.

Building on the best condition obtained after 26 human-guided trial-and-error depositions (Figure 1), we next applied Bayesian optimization to accelerate the process optimization of FePt-BN granular films. The initial dataset consisted of these 26 process conditions – namely the deposition rates of FePt-25 at.% BN and FePt-55 at.% BN targets, the film thickness, and the N_2_ gas concentration – together with the corresponding microstructural and magnetic metrics (*D*_1_ and *μ*_0_*H*_c_). In each optimization cycle, the trained surrogate model generated on the order of ~10^2^ candidate predictions and identified a small set of Pareto-optimal solutions (typically ~8) that represent trade-offs between the target objectives (e.g. maximizing *μ*_0_*H*_c_ while reducing *D*_1_; see schematic example in graphical abstract). From the Pareto set obtained in the first cycle, we selected six promising recipes for experimental validation; these results were then appended to the dataset and used to perform a second Bayesian optimization cycle, from which three additional Pareto solutions were tested. After incorporating these new measurements, a third cycle was conducted, and the most promising Pareto solution was experimentally realized; the resulting microstructure and magnetic properties are summarized in Figure 9. The final condition achieved *μ*_0_*H*_c_ = 2.38 T with *D*_1_ = 5.98 nm, corresponding to an areal grain density of 12.68 Tgrains/in^2^ and an order parameter *S* = 0.64 (Figure 9). Compared with the best outcome obtained by the initial 26 trial-and-error experiments (*μ*_0_*H*_c_ ≈ 2.1 T and *D*_1_ ≈ 6.4 nm in Figure 1), Bayesian optimization delivered a more desirable combination-higher *μ*_0_*H*_c_ and smaller grains, using only 10 additional experiments. These results demonstrate that Bayesian optimization can effectively guide process optimization in FePt-BN granular media, reducing the need for exhaustive parameter sweeps and minimizing reliance on intuition while converging rapidly toward improved performance.

**Figure 9.**
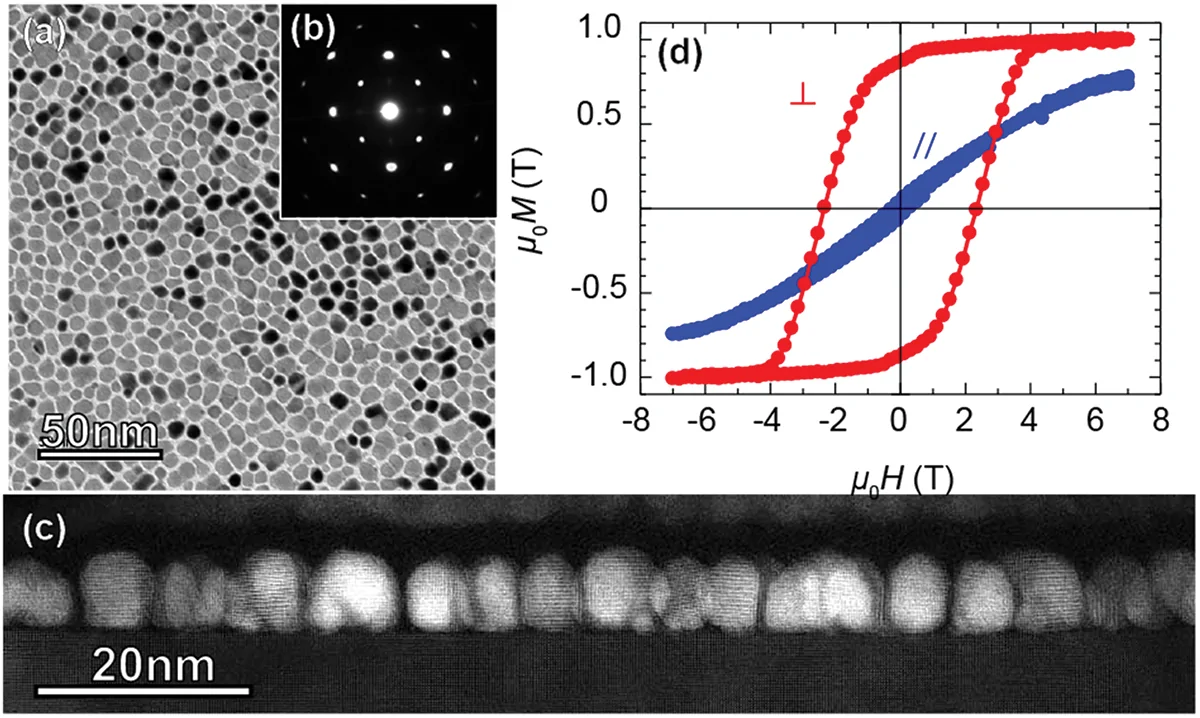
(a) Plan-view TEM image, (b) corresponding selected-area electron diffraction (SAED) pattern, (c) cross-sectional TEM image, and (d) out-of-plane and in-plane magnetization hysteresis loops of FePt-BN granular film fabricated using the most promising Pareto solution identified after three cycles of Bayesian optimization. The optimized sample exhibits a *μ*_0_*H*_c_ of 2.38 T, an average grain diameter (*D*_1_) of 5.98 nm, an areal grain density of 12.68 Tgrains/in^2^.

## 4. Conclusion

We investigated FePt-BN granular thin films as HAMR media, focusing on microstructural and *μ*_0_*H*_c_ optimization. Initial trial-and-error approaches yielded FePt-BN media with an in-plane grain diameter of 6.4 nm, a *μ*_0_*H*_c_ of 2.1 T, a pitch distance of 6.9 nm, an areal density of 9.9 Tgrains/in^2^, and L1_0_ order parameter (*S*) of 0.97. The optimal condition identified by subsequent Bayesian optimization – a condition unlikely to be reached by human intuition yet found with only a modest number of additional experiments – yielded a higher *μ*_0_*H*_c_ (2.38 T) and smaller grain diameter (5.98 nm), corresponding to an areal density of 12.68 Tgrains/in^2^ and *S* = 0.64.

Adopting that PCA of XRD and TEM data can serve as an efficient alternative to traditional, labor-intensive phase quantification methods (such as Rietveld analysis). In particular, the primary PCA score (PC01) from XRD was found to be strongly correlated with the L1_0_-FePt phase fraction and ordering, highlighting the broader utility of PCA-based feature extraction for rapid, quantitative assessment of correlation between *μ*_0_*H*_c_ and L1_0_ order/microstructure in high-throughput or data-driven materials development.

Furthermore, our study demonstrates that a machine-learning workflow using random forest regression allowed us to efficiently identify key process parameters – especially L1_0_ crystallinity, BN fraction, and features from XRD and TEM data – that critically influence *D*_1_, *D*_2_ and *μ*_0_*H*_c_. We found that *μ*_0_*H*_c_ is not solely determined by *D*_1_, *D*_2_ and *S* but is more strongly correlated with microstructural features described by principal-component–based descriptors, including those extracted from XRD and TEM data, such as PC01. These mixed descriptors capture not only *D*_1_, *D*_2_ and *S*, but also microstructural inhomogeneities, such as grain isolation, sub-grain structure, and interfacial contrast, which collectively govern *μ*_0_*H*_c_. Linear correlation analysis indicated that parameters such as ‘Roundness’ and *D*_2_ also exhibit relatively strong correlations with *μ*_0_*H*_c_, reflecting the importance of grain isolation and fine-scale structure beyond simple grain size.

These results demonstrate that machine learning-guided process optimization is a powerful tool for the accelerated development of advanced FePt-BN granular HAMR media, enabling the design of media with superior recording performance. Our work also highlights the broader potential of integrating combinatorial materials processing with established machine-learning techniques in an active-learning framework to unlock new functionalities and accelerate innovation in data storage technologies, supporting future ultrahigh-density hard disk technologies.

## Supplementary Material

Supplemental Material
